# Multi‑label classification of biomedical data

**DOI:** 10.3892/mi.2024.192

**Published:** 2024-09-09

**Authors:** Io Diakou, Eddie Iliopoulos, Eleni Papakonstantinou, Konstantina Dragoumani, Christos Yapijakis, Costas Iliopoulos, Demetrios A. Spandidos, George P. Chrousos, Elias Eliopoulos, Dimitrios Vlachakis

**Affiliations:** 1 Laboratory of Genetics, Department of Biotechnology, School of Applied Biology and Biotechnology, Agricultural University of Athens, 11855 Athens, Greece; 2 University Research Institute of Maternal and Child Health and Precision Medicine, National and Kapodistrian University of Athens, ‘Aghia Sophia’ Children's Hospital, 11527 Athens, Greece; 3 School of Informatics, Faculty of Natural and Mathematical Sciences, King's College London, London WC2R 2LS, UK; 4 Laboratory of Clinical Virology, School of Medicine, University of Crete, 71003 Heraklion, Greece

**Keywords:** myocardial infarction, multi-label classification, biomedical datasets, label graph, precision medicine, complication prediction

## Abstract

Biomedical datasets constitute a rich source of information, containing multivariate data collected during medical practice. In spite of inherent challenges, such as missing or imbalanced data, these types of datasets are increasingly utilized as a basis for the construction of predictive machine-learning models. The prediction of disease outcomes and complications could inform the process of decision-making in the hospital setting and ensure the best possible patient management according to the patient's features. Multi-label classification algorithms, which are trained to assign a set of labels to input samples, can efficiently tackle outcome prediction tasks. Myocardial infarction (MI) represents a widespread health risk, accounting for a significant portion of heart disease-related mortality. Moreover, the danger of potential complications occurring in patients with MI during their period of hospitalization underlines the need for systems to efficiently assess the risks of patients with MI. In order to demonstrate the critical role of applying machine-learning methods in medical challenges, in the present study, a set of multi-label classifiers was evaluated on a public dataset of MI-related complications to predict the outcomes of hospitalized patients with MI, based on a set of input patient features. Such methods can be scaled through the use of larger datasets of patient records, along with fine-tuning for specific patient sub-groups or patient populations in specific regions, to increase the performance of these approaches. Overall, a prediction system based on classifiers trained on patient records may assist healthcare professionals in providing personalized care and efficient monitoring of high-risk patient subgroups.

## Introduction

Machine learning is a subset of artificial intelligence, aimed at developing ‘intelligent’ algorithms that harness data to execute tasks with optimal performance (1). Machine learning algorithms can be broadly split into four categories: Supervised, semi-supervised, unsupervised and reinforcement-learning (2). In supervised learning, the algorithm is given a dataset known as ‘training data’, where each training sample corresponds to one or several inputs and the desired output (3). Through an iterative process, the algorithm determines a function which can correctly predict the desired output from a set of new, previously unknown inputs. Supervised machine-learning tasks include classification, regression and forecasting (4). Unsupervised learning, conversely, is carried out on unlabeled datasets with the aim of extracting patterns and information without external supervision (5). Semi-supervised learning, as indicated by the name, falls somewhere between supervised and unsupervised techniques, as only a portion of the training data is labeled (6). Lastly, during reinforcement learning tasks, an intelligent agent takes actions in a set environment (7). The actions return a reward, while also influencing the environment and the state of the agent. The goal of the agent is to ‘learn’ the policy which maximizes the reward function, or more generally, maximizes the reinforcement signal that is generated by the rewards (8).

Machine learning approaches, as a whole, have become increasingly relevant in the current era of ‘big data’. Data technologies are rapidly evolving, with data storage sizes entering petabytes, cloud services enabling high data transfer speed and computational systems shifting towards high performance cluster computing (9-11). This has allowed the implementation of machine learning in a range of diverse fields, including healthcare. A trove of biomedical data is generated daily throughout the process of medical practice and patient care. Examples of such data include imaging results (ultrasounds, magnetic resonance imaging, computed tomography scans), laboratory test results (cell cultures, biological material analyses and sequencing), patient medical history, drug effects and interactions and patient health outcomes (12). These can be regarded as attractive targets for the implementation of machine learning algorithms, wherein the desired objective is tailored to the respective challenge.

The prediction of disease complications constitutes a key aspect of patient treatment and management (13). The study published in 2022 by Ghosheh *et al* (14) investigated the use of a predictive system to predict the risk of developing complications in patients diagnosed with coronavirus disease 2019 (COVID-19), trained on data of >3,000 patients in the United Arab Emirates. The prediction of disease complications, overall, can be interpreted as a predictive classification problem, thus opening the door to the application of supervised machine learning algorithms. The current status of the patient can be analyzed by diagnostic classification models, while potential outcomes can be predicted by prognostic classification models (15). Predictive classification algorithms have been implemented in the context of various diseases within the past years. During the peak of the recent severe acute respiratory syndrome coronavirus 2 (SARS-CoV-2) pandemic, classification models were designed to tackle various aspects of the disease, such as the detection of viral infection through X-ray imaging and CT scans or the prediction of outcomes of patients with COVID-19 using their recorded characteristics as input (16-18). This is even more important under the scope of personalized medicine, as the individual patient profile, which includes features, such as comorbidities, age, ethnic background etc., may affect disease progression and clinical manifestations (19). In a number of cases, several conditions, or labels, may be assigned to a single patient; for example, an individual may be suffering from COVID-19, while also exhibiting cardiovascular issues and high cholesterol. In such a case, the challenge of building predictive models can be regarded as a multi-label classification problem.

This type of classification task falls under the supervised machine learning ‘umbrella’ and constitutes a modeling problem where the class, also known as target or label, is predicted for a data point, otherwise known as input sample (20). In single-label classification, from a collection of discrete *L* (*L*>1) labels, a single label *‘l’* is assigned to each input sample (21). If the number of labels *L* is 2, the task is considered a binary classification problem, whereas if the number of labels *L*>2, it is considered a multiclass classification problem (22). In the case where each input sample is associated with a set of non-exclusive class labels, the task is called multi-label classification (23).

One of the common challenges in classification and even more so in the case of multi-label, biomedical datasets, is the presence of imbalanced data (24). In the multi-label setting, imbalance can be traced to three distinct levels, imbalance within labels, imbalance between labels and imbalance within label sets (25). In inter-label imbalance, the label column contains a disproportionate ratio of negative vs. positive samples, effectively obscuring the signal of the particular label (26). In intra-label imbalance, there is difference in frequency between labels, with frequency most often termed as the number of positive instances (27). Labels with an abundance of positive samples are considered in majority, while the rest are minority labels (28). In multi-label classification, there is a possibility for a sample to simultaneously contain a label in minority and another label that is in majority. Labels in majority are termed head labels and labels in minority tail label (29). The last source of multi-label imbalance is the existence of label sets, where some sets may be more frequent in the dataset than others (30-32).

Myocardial infarction (MI), or more generally known as a ‘heart attack’, is caused by a decrease or total interruption in blood flow to part of the myocardium (33). As per the World Health Organization, more than four out of five cardiovascular disease-related deaths are due to heart attacks and strokes, while a third of these deaths concern individuals aged <70 years (34). Thrombosis has been established as the most prominent driver of acute MI, itself stemming from events of atherosclerosis and inflammation (35). Atherosclerotic lesions emerge as thickening of the coronary inner walls of the artery or as fatty streaks, ultimately leading to thrombus formation (36). During their hospitalization, patients with acute MI often face severe complications, such as shock, stroke, atrioventricular block, respiratory failure and cardiopulmonary arrest (37-39). Shock and posterior cerebral artery, in particular, constitute prevalent causes of mortality in patients with acute MI (40). Establishing a robust plan of action is inextricably linked to the successful management of MI-related complications in the hospital setting. Therefore, the application of a classification model to predict potential MI complications may prove useful in informing the decision-making of medical professionals.

In the present study, to demonstrate the potential of leveraging biomedical patient data in the context of predictive classification, a multi-label classification model was trained on a recently released, public dataset of MI patient data.

## Data and methods

### Data collection and preprocessing

**Figure 1:**
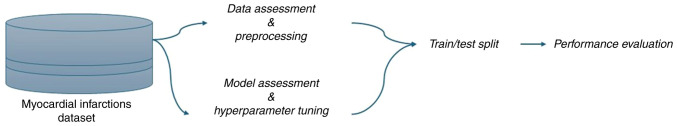
Overview of the steps of the pipeline used herein.

Machine-learning tasks require, first and foremost, a solid data-related foundation, for the implementation and validation of the framework and its constituent algorithms. To explore the multi-label classification task, a public dataset regarding the outcomes of patients with MI was retrieved (41). The original dataset consists of 114 descriptive metrics collected for a total of 1,700 patients with MI, along with information regarding patient outcomes and complications, split into 12 candidate categories. Descriptive analytics of the dataset and information regarding the convention of column names can be found in Table SI. The recorded patient metrics are of mixed nature, falling into one of the following types: Binary, real and ordinal. The binary data stems from two attribute-handling approaches during the dataset's original creation. First, categorical variables were dummy coded into 0 and 1; for example, the column ‘SEX’ where values ‘female’ and ‘male’ were arbitrarily encoded to 0 and 1, respectively. Secondly, binary encoding was used to indicate presence or absence of attributes, such as in the ‘symptomatic hypertension’ (SIM_GIPERT) column, where presence or absence was encoded to 1 and 0, respectively. The real, or numerical data, within the dataset mainly concern patient measurements taken during assessment by the medical professionals and throughout hospitalization, such as ‘systolic blood pressure according to Emergency Cardiology Team’ (S_AD_KBRIG). Lastly, the ordinal feature data contained values with intrinsic order; for example, the column ‘presence of essential hypertension’ (GB), which took values 0, 1, 2 or 3, corresponding to absence, stage 1, stage 2 and stage 3 essential hypertension. An overview of the steps of the pipeline is presented in Fig. 1.

As per the dataset's curators, there are four candidate time settings for the construction of the prediction challenge: the time of admission to hospital, 24, 48 and 72 h following hospital admission. The selection of time setting determines the columns which can be used as input data during model fitting. The end of the first day (24 h post-hospital admission), was selected as the time setting for the present implementation of the classification algorithm, leading to the exclusion of six input columns, plus the patient ID column which serves no predictive purpose.

Missing data constitutes a common issue in biomedical datasets, as the process of data collection is error-prone, particularly in a hospital setting where such processes are rarely automated and are most often handled by the doctors and nurses themselves. Discarding every single sample/patient record that is missing a portion of the input features could harm the potential of the classifier, as it would markedly lessen the amount of information available to train the classifier on. A common strategy to remedy this problem is data imputation, where the missing data are imputed by various methods. In the present pipeline, input columns which contained missing values above a threshold of 85% of the total number of rows were removed, and a multivariate imputer was used to estimate the missing values in the rest of the dataset. Lastly, a baseline for the type of target data was set. As aforementioned, the output (target) data span columns 113-124 of the original dataset. All columns but one contained data in binary form, denoting presence or absence of a complication/outcome. To maintain the binary type uniform across the output data, the singular column ‘lethal outcome’ (LET_IS), which contained non-ordinal, numerical data, was one-hot encoded. To one-hot encode a variable with *n* possible, non-ordinal values, *n* separate columns are generated and the presence or absence of the value in a sample is denoted by 1 and 0, respectively. In the case at hand, numbers 0-7 had been assigned to the outcomes ‘alive’, ‘cardiogenic shock’, ‘pulmonary edema’, ‘myocardial rupture’, ‘progress of congestive heart failure’, ‘thromboembolism’, ‘asystole’, ‘ventricular fibrillation’, and were subsequently split into eight separate binary data columns. The final dataset can be found in Table SII. Additionally, a detailed description of the original MI database, descriptive statistics and a list of the column abbreviations can be found via the following weblink: 10.25392/leicester.data.12045261.v3.

### Label relations exploration

In classification tasks, it is useful to explore the label space and the associations between the labels reported within the dataset. Graphs are increasingly used in the study of complex systems, such as protein or traffic networks, enabling the implementation of embedding algorithms (42). In the multi-label setting, graphs represent multiple levels of information, as graph edges could reflect a range of relationships between labels, from simpler to more complex ones (43). Furthermore, the study of clustering and interactions between labels could elucidate more obscure factors underlying the network's structure (44). For example, a network of comorbidities represented as a multi-label graph could provide insight into subtle interplays between pathological conditions.

To explore the graph space, the Label Cooccurrence Graph Builder class was imported from the skmultilearn.cluster module as the graph builder base (45). The NetworkXLabelGraphClusterer class from the same module was used to study the community and clustering trends across the label instances by use of the Louvain method (46), and NetworkX was used to visualize the graph (47).

### Data-driven model selection and hyperparameter tuning

The selection of a classification algorithm is entirely dependent on the nature of the multioutput problem concerned, and there is no established guideline for choosing a model. Two factors to be considered during the selection process are performance and efficiency. Efficiency is inherently tied to model aspects, such as its scalability, the type of label combinations within the dataset, and so on. A classification algorithm that exhibits high performance may suffer from low efficiency; for example, choosing a model that trains a single classifier per label would be unsuitable or too slow for a task with a large label space. Performance may be viewed as the model's generalization capability and there exist several evaluation metrics that can be employed. Precision measures the model's reliability in classifying a sample as positive, accuracy measures how well the model performs across all the classes of the dataset (48) and recall represents the ratio of how many of the actual labels were correctly predicted (49). While the aforementioned metrics hold up well in cases of multi-class classification, in multi-label classification, where the model's predictions can range from fully or partially correct to fully incorrect, the adjustment of evaluation measures is required to reflect these subtleties (50). Hamming loss (HL) measures the hamming distance between the true and the predicted label, penalizing the incorrectly predicted labels in a predicted label set individually; therefore, the metric is capable of reflecting the notion of partially correct model predictions (23). The metric ranges from 0 to 1 and the lower the HL, the better performance is exhibited by the multi-label classification model.

To carry out data-driven model selection, a set of algorithms were trained on the dataset and their performance was evaluated by the HL metric. Parameters which control the model's architecture are termed ‘hyperparameters’ and the process of exploring possible choices towards an optimal model architecture is termed ‘hyperparameter tuning’ (51). To carry out the step, aa cross-validated grid search was employed. The method, which is available through the GridSearchCV class of the scikit-learn module, entails an exhaustive search over a parameter grid, in order to yield optimal model parameters (52). Inputting an integer for the ‘cv’ parameter of the class enables a stratified k-fold split, where the dataset is divided into k partitions and for each split, a search through a user-set range of the hyperparameter spaces is executed, fitting and scoring each combination to elect the hyperparameters which lead to the best model performance (53). The pool of candidate classification algorithms contained the following: Classifier Chains (CC) with Random Forest as the base classifier, Classifier Chains with XGBoost as the base classifier, Binary Relevance k-Nearest Neighbors (BRkNN) Classifier, Random Forest (RF) Classifier, Multi-label k-Nearest Neighbors Classifier (MLkNN) and OneVsRest with XGboost, all of which are available through the scikit-learn and XGBoost libraries (54,55).

Extreme Gradient Boosting (XGBoost) is an advanced implementation of the Gradient Boosting decision tree algorithm (55). Gradient Boosting builds the first learner, a decision tree, on the training dataset to perform the prediction of the target samples, then calculates the loss, which is the difference between the true value (or true label) and the predicted value that has been generated from the first learner (56). The residual of the loss function is calculated using the Gradient Descent Method and is used as the target variable for the next iteration, where an improved learner is built (57). In brief, numerous models are trained sequentially, and the algorithm aims to boost them into a strong learner which best predicts the target. XGBoost implements parallel processing, increasing the algorithm's speed ten-fold, compared to standard Gradient Boosting (55). Furthermore, the algorithm is flexible, allowing the user to select custom optimization objectives and fine-tune booster and task parameters. XGBoost does not naively support multi-output classification, hence, to implement extreme gradient boosting in the multi-label classification problem, the XGBoost model was wrapped inside the MultiOutputClassifier class from the scikit-learn module (54).

### Training and K-fold cross validation

In traditional machine-learning model development, the model is trained on a partition of the data, called the training set, and a set of the data unseen to the model during training is used as the test set, to evaluate the performance of the algorithm based on new data (58,59). K-fold cross validation entails dividing the dataset into k non-overlapping groups of rows, then training the machine-learning model on all the groups save for a hold-out fold, which is then used as the test set (60). The process is repeated across all folds, until each fold has been used as the hold-out test set, and model performance is averaged across the folds (61). To carry out k-fold cross validation and account for the imbalanced multi-label dataset, high-order iterative stratification was implemented via the scikit-learn ‘iterative_stratification’ module (62,63). In brief, dataset splits are created while maintaining balanced representation of labels within each fold as much as possible. To evaluate model performance on the imbalanced dataset, HL was selected as the evaluation metric. Building, training and testing the classifiers was carried out in a Jupyter environment, using a 4-core CPU and 2-core GPU-accelerated system.

## Results

**Figure 2:**
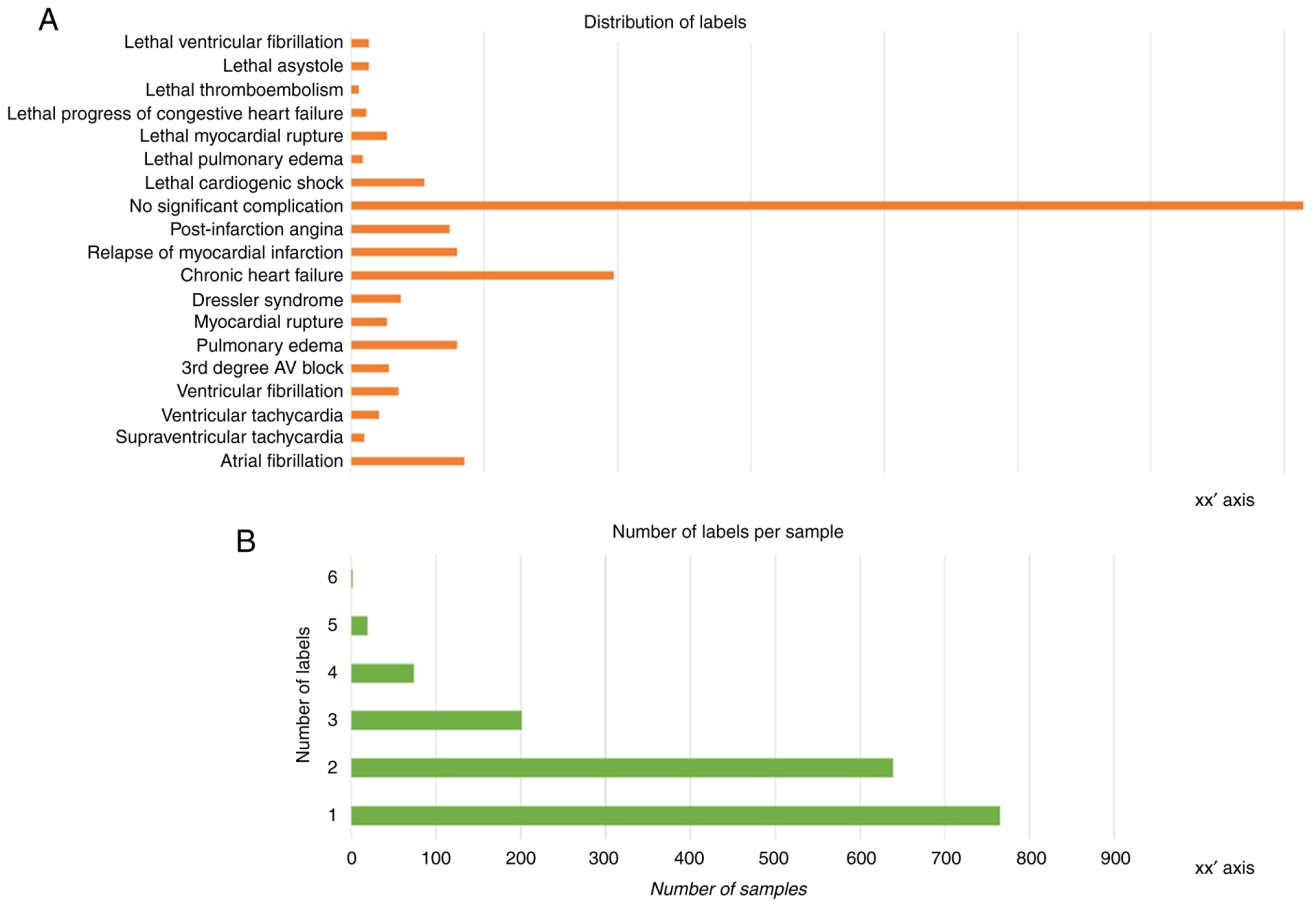
(A) Bar histogram depicting the distribution of label instances across the dataset. (B) Bar histogram representing the distribution of total label numbers assigned to each sample (a patient). Note that the number of labels exhibited by a patient will normally be smaller than the total of possible outcomes, as one patient will not exhibit all possible outcomes.

At the end of the data preprocessing steps, the dataset contained 1,700 rows corresponding to 1,700 patient samples, 100 columns of patient measurements/features serving as the input (X) data and 18 columns of patient outcomes, serving as the target label (y) data. As visible in the histogram plot illustrated in Fig. 2A, there is notable imbalance across the instance of labels. The number of labels per sample is also varied, with most samples assigned one or two labels and very few samples exhibiting more than three labels (Fig. 2B). This can be traced back to the challenge of data collection that was touched upon in the introduction segment; the limited number of patients affects the number of labels (outcomes) that happened to be present among them and were thus recorded.

**Figure 3:**
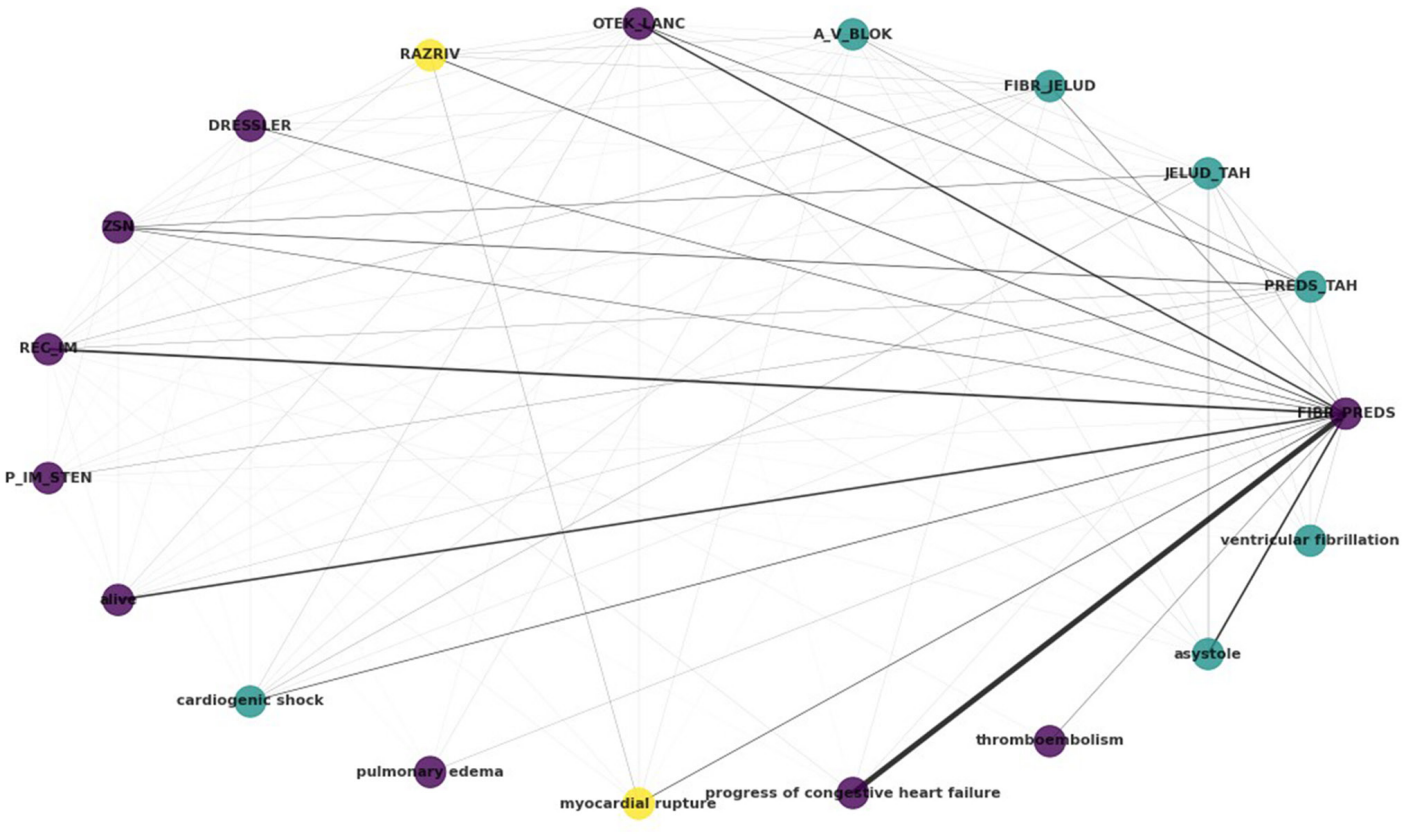
Label graph of the labels represented as nodes, with the thickness of edges corresponding to co-occurrence rate. By using the Louvain algorithm, three clusters are reported, denoted by purple, yellow and light blue colour. Graph nodes sharing a colour belong to the same cluster. The nodes with the same colour have been assigned to the same cluster.

The output label portion of the dataset was used as input to generate information regarding label interactions and relationships. In the case at hand, the potential outcomes and complications of hospitalized patients with MI are regarded as labels. The results of the exploration of the label space can be summarized in the circular graph of Fig. 3. Each label, i.e., each complication, is represented as a node in the graph, with an edge existing when there is co-occurrence between the labels, weighted by the frequency of co-occurrence. By using the Louvain algorithm, a popular community detection method, three clusters are reported, denoted by purple, yellow and light blue colour.

The *atrial fibrillation (FIBR_PREDS)* complication (non-lethal) exhibits strong relations with *progress of congestive heart failure* and *asystole*, both tagged within the dataset as lethal complications. Notable relations are also reported between *atrial fibrillation*-*relapse of the myocardial infarction*, and *atrial fibrillation*-*pulmonary edema (OTEK_LANC)*. It is also noteworthy that, as regards lethal outcomes, *cardiogenic shock*, *asystole* and *ventricular fibrillation* have been clustered together, as have *pulmonary edema*, *progress of congestive heart failure* and *thromboembolism*. The lethal complication of *myocardial rupture*, on the other hand, has been clustered with- and exhibits relations with-the *myocardial rupture (RAZRIV)* complication label, potentially indicating that patients with myocardial rupture were assigned to both labels up to the lethal outcome.

**Figure 4:**
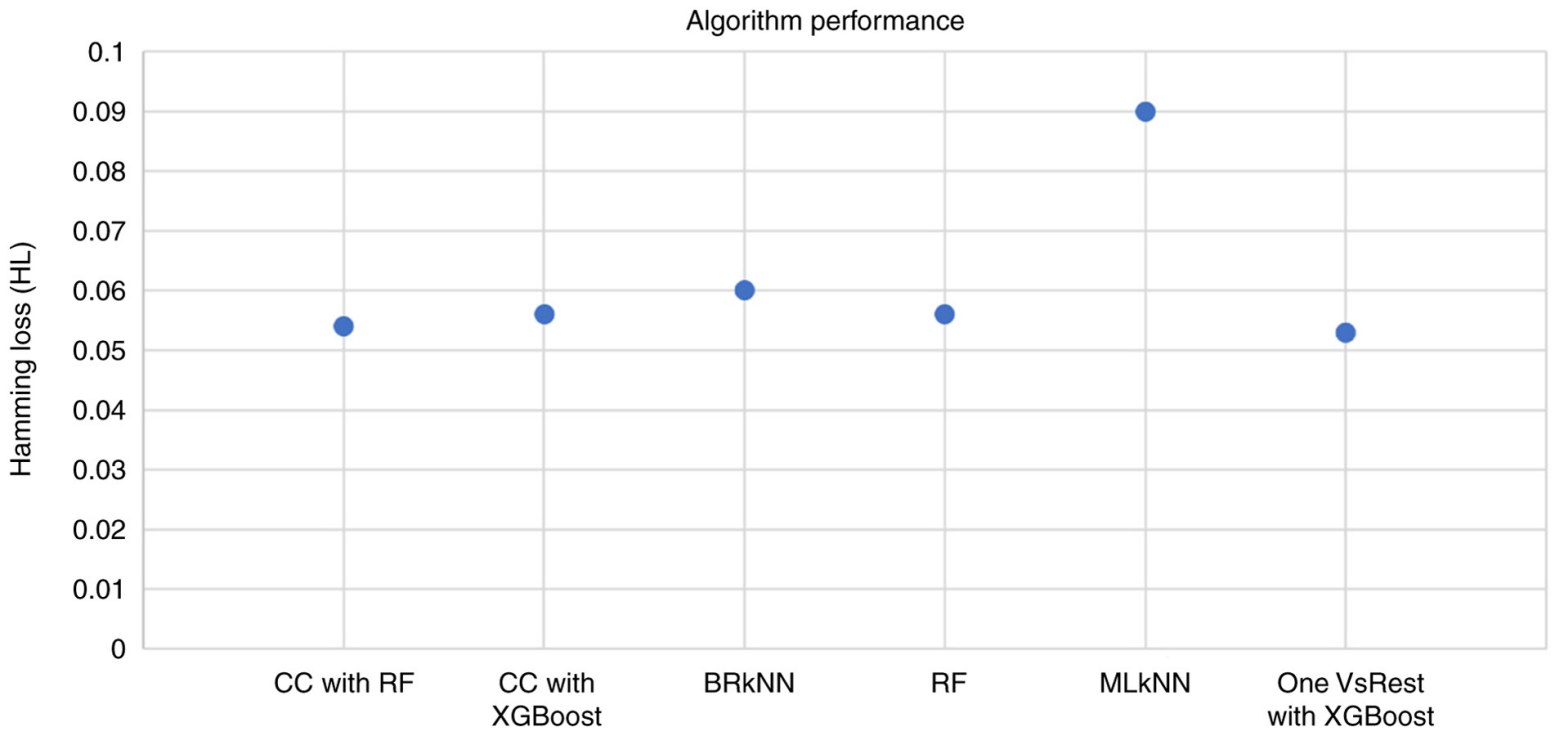
Chart reflecting the candidate classification algorithms and their respective performance as measured through the hamming loss metric. CC, Classifier Chains; BRkNN, Binary Relevance k-Nearest Neighbor; RF, Random Forest; MLkNN, Multi-label k-Nearest Neighbor.

The candidate algorithms were evaluated according to their performance on the dataset and the results are summarized in Fig. 4. The highest HL was exhibited by the Multi-label k-Nearest Neighbor (MLkNN) classifier, whereas the lowest HL (0.053) was exhibited by the OnevsRest classifier with XGBoost. This intuitive classifier strategy, also known as the one-vs.-all, trains a binary classifier independently for each label, and can be applied to both multi-class and multi-label problems.

## Discussion

Predictive classification algorithms have been implemented in the context of various diseases over the past years. During the peak of the recent SARS-CoV-2 pandemic, classification models were designed to tackle various aspects of the disease, such as the detection of viral infection through X-ray imaging and CT scans or the outcome prediction of COVID-19 patients using their recorded characteristics as input (16-18). This is even more critical under the scope of personalized medicine, as the individual patient profile, which includes features, such as comorbidities, age, ethnic background etc., may affect disease progression and clinical manifestations (19). In a number of cases, several conditions, or labels, may be assigned to a single patient; for example, an individual may be suffering from COVID-19, while also exhibiting cardiovascular issues and high cholesterol levels. In such a case, the challenge of building predictive models can be regarded as a multi-label classification problem, as explored herein.

The development and testing of classifiers is a complex task, particularly in the case of multi-label classification. The comparative evaluation of various algorithms on the myocardial infarction dataset elected the OneVsRest heuristic as the best-performing one. Furthermore, the use of XGBoost as the base classifier enabled the fine-tuning of the model and accelerated the learning process. XGBoost was identified as the best performing algorithm in a study published in 2022 for a similar predictive task. That study aimed to develop and validate a machine learning-based model to predict regional lymph node metastasis in osteosarcoma using data from 1,201 patients, identifying T and M stage, surgery and chemotherapy as significant risk factors and XGBoost as the best performing predictive algorithm for that task (64).

The use of disease datasets is also employed by other frameworks; for example, Tang *et al* (65) described a Gaussian randomizer-based system for early fundus screening with privacy preserving and domain adaptation, employing a multi-disease dataset.

It would be of interest, as an extension of the proposed framework, to evaluate a set of different base classifiers in the context of the OneVsRest strategy and observe the effect that their substitution may have on the classification performance. The present study focused on a subset of the available algorithms and strategies; therefore, there exist other potential machine-learning components and techniques to be evaluated on this task. In terms of the dataset itself, graph exploration highlighted shared label instances that potentially contain information relevant to myocardial infarction pathophysiology. The label imbalance that marks the dataset constitutes an interesting point in terms of handling a multi-label classification problem. Methods to address imbalanced datasets in multi-label classification have been reviewed elsewhere and include, but are not limited to, random oversampling and undersampling, heuristic oversampling, cost-sensitive learning, and ensemble approaches (26,31). Oversampling is the process of increasing the rate of minority class instances within an imbalanced dataset to compensate for the occurrence of common classes (66). Modern and widely-used techniques, such as the Synthetic Minority Over-sampling Technique (SMOTE) create synthetic data points by using the feature space of the minority class and k-nearest neighbors; however, applying the k-nearest neighbor approach on binary input data such as the dataset at hand would serve no purpose (67). Furthermore, the existence of binary input data excludes the use of SMOTENC, the SMOTE extension for numerical and categorical features (67). Therefore, if an added oversampling step were to be applied, a custom oversampling function would need to be created to increase tail label samples based on the calculated oversampling ratio of the labels. Lastly, the concept of errors constitutes an important facet of developing accurate and reliable biomedical classification models. A classifier is subject to two main types of errors, false positives, also known as type I errors, and false negatives, also known as type II errors (59). In the case of false positives, the classifier predicts a label which is not present in the test set, while in the case of false negatives, a label that should have been predicted is missing. Similarly, true positives are results where the classifier has correctly predicted a label presence and in true negatives, the classifier has correctly predicted the absence of label, i.e., the absence of the positive instance. In the case of disease complication predictions, we are greatly invested in limiting the rate of false negatives, where the classifier fails to predict a label (a complication) which in truth exists. One could argue that in the hospital setting, it would be less damaging to monitor a patient in anticipation of a complication that turns out to be a false positive, than failing to catch a complication that may be lethal. Therefore, the selection of performance metrics and the penalties enforced on the errors of the model are greatly affected by the nature of the disease which we wish to interpret as a classification task.

In conclusion, MI constitutes a highly frequent phenomenon in the subset of the population suffering from cardiovascular problems. The development of accurate and scalable systems to support the decision-making process of the medical professionals in the hospital can alleviate the weight of patient management and may potentially increase the odds of survival for myocardial infarction patients. The use of predictive systems for disease-related challenges has been garnering attention the past years with the increase in computational power and novelty of algorithms.

The data-driven approach presented herein and the obtained results underline the potential of machine learning applications in risk predictions, in particular for the challenge associated with MI. As demonstrated through the evaluation, high-performance algorithms, such as the Extreme Gradient Boosting algorithm can be employed as base classifiers in the context of machine-learning model development, while disciplines such as graph theory can shed light into the elaborate networks underlying myocardial infarction progression. Public dataset repositories can provide the large-scale quantities of biomedical and patient data that are required to build efficient and reliable predictive classification models. This data-driven approach can be further scaled and enhanced; there exists promise in the use of ensemble models, made up of different classifiers with different aptitude towards predicting specific labels. Overall, the classifier-based pipeline holds the potential to support the decision-making process of healthcare professionals and aid a proactive approach to patient care.

## Supplementary Material

Descriptive analytics of the dataset and information regarding the convention of column names.

Details of the final dataset.

## Data Availability

The code samples and raw data analyzed during the present study can be found at: https://github.com/IoDiakou/MLC-on-biomedical-data.git and http://darkdna.gr.

## References

[1] Anderson JR

[2] Russell S, Norvig P

[3] Somani P, Kaur G (2020). A review on supervised learning algorithms. Int J Adv Sci Technol.

[4] Singh P

[5] Gentleman R, Carey VJ

[6] Hady MFA, Schwenker F

[7] Sutton RS, Barto AG

[8] Lee D, Seo H, Jung MW (2012). Neural basis of reinforcement learning and decision making. Annu Rev Neurosci.

[9] Czarnul P, Proficz J, Krzywaniak A (2019). Energy-aware high-performance computing: Survey of state-of-the-art tools, techniques, and environments. Sci Program.

[10] Mascetti L, Arsuaga Rios M, Bocchi E, Vicente JC, Cheong BCK, Castro D, Collet J, Contescu C, Labrador HG, Iven J (2020). CERN disk storage services: Report from last data taking, evolution and future outlook towards Exabyte-scale storage. EPJ Web Conf.

[11] Amin R, Vadlamudi S, Rahaman MM (2021). Opportunities and challenges of data migration in cloud. Eng Int.

[12] Dash S, Shakyawar SK, Sharma M, Kaushik S (2019). Big data in healthcare: Management, analysis and future prospects. J Big Data.

[13] Wachter RM

[14] Ghosheh GO, Alamad B, Yang KW, Syed F, Hayat N, Iqbal I, Al Kindi F, Al Junaibi S, Al Safi M, Ali R (2022). Clinical prediction system of complications among patients with COVID-19: A development and validation retrospective multicentre study during first wave of the pandemic. Intell Based Med.

[15] van Smeden M, Reitsma JB, Riley RD, Collins GS, Moons KG (2021). Clinical prediction models: Diagnosis versus prognosis. J Clin Epidemiol.

[16] de Souza FSH, Hojo-Souza NS, dos Santos EB, da Silva CM, Guidoni DL

[17] Ezzoddin M, Nasiri H, Dorrigiv M

[18] Pathak Y, Shukla PK, Tiwari A, Stalin S, Singh S (2022). Deep transfer learning-based classification model for COVID-19 disease. IRBM.

[19] Yuan B (2021). Towards a clinical efficacy evaluation system adapted for personalized medicine. Pharmgenomics Pers Med.

[20] Kotsiantis SB, Zaharakis ID, Pintelas PE (2006). Machine learning: A review of classification and combining techniques. Artif Intell Rev.

[21] Wei Y, Xia W, Huang J, Ni B, dong J, Zhao Y, Yan S

[22] Soofi AA, Awan A (2017). Classification techniques in machine learning: Applications and issues. J Basic Appl Sci.

[23] Tsoumakas G, Katakis I (2009). Multi-label classification: An overview. Int J Data Warehous Min.

[24] Herrera F, Charte F, Rivera AJ, del Jesus MJ

[25] Sun Y, Wong AKC, Kamel MS (2009). Classification of imbalanced data: A review. Int J Pattern Recognit Artif Intell.

[26] Tarekegn AN, Giacobini M, Michalak K (2021). A review of methods for imbalanced multi-label classification. Pattern Recognit.

[27] Charte F, Rivera AJ, del Jesus MJ, Herrera F (2019). Dealing with difficult minority labels in imbalanced mutilabel data sets. Neurocomputing.

[28] Charte F, Rivera A, del Jesus MJ, Herrera F

[29] Huang Y, Giledereli B, Köksal A, Ozgur A, Ozkirimli E

[30] Giraldo Forero AF, Jaramillo-Garzón J, Ruiz-Muñoz J, Castellanos-Dominguez G

[31] Tahir MA, Kittler J, Bouridane A (2012). Multilabel classification using heterogeneous ensemble of multi-label classifiers. Pattern Recognit Lett.

[32] Cao P, Liu X, Zhao D, Zaiane O

[33] Saleh M, Ambrose JA (2018). Understanding myocardial infarction. F1000Res.

[35] Badimon L, Vilahur G (2014). Thrombosis formation on atherosclerotic lesions and plaque rupture. J Intern Med.

[36] Asada Y, Yamashita A, Sato Y, Hatakeyama K (2018). Thrombus formation and propagation in the onset of cardiovascular events. J Atheroscler Thromb.

[37] Shavadia JS, Chen AY, Fanaroff AC, de Lemos JA, Kontos MC, Wang TY (2019). Intensive care utilization in stable patients with ST-segment elevation myocardial infarction treated with rapid reperfusion. JACC Cardiovasc Interv.

[38] Abrignani MG, Dominguez LJ, Biondo G, Di Girolamo A, Novo G, Barbagallo M, Braschi A, Braschi G, Novo S (2005). In-hospital complications of acute myocardial infarction in hypertensive subjects. Am J Hypertens.

[39] Malla RR, Sayami A (2007). In hospital complications and mortality of patients of inferior wall myocardial infarction with right ventricular infarction. JNMA J Nepal Med Assoc.

[40] Babaev A, Frederick PD, Pasta DJ, Every N, Sichrovsky T, Hochman JS (2005). Trends in management and outcomes of patients with acute myocardial infarction complicated by cardiogenic shock. JAMA.

[41] Golovenkin SE, Gorban A, Mirkes E, Shulman VA, Rossiev DA, Shesternya PA, Nikulina SY, Orlova YV, Dorrer MG

[42] Yang J, Leskovec J (2015). Defining and evaluating network communities based on ground-truth. Knowl Inf Syst.

[43] Huang SJ, Zhou ZH (2021). Multi-label learning by exploiting label correlations locally. Proc AAAI Conf Artif Intell.

[44] Chakravarty A, Sarkar T, Ghosh N, Sethuraman R, Sheet D (2020). Learning decision ensemble using a graph neural network for comorbidity aware chest radiograph screening. Annu Int Conf IEEE Eng Med Biol Soc.

[45] Szymański P, Kajdanowicz T, Kersting K (2016). How is a data-driven approach better than random choice in label space division for multi-label classification?. Entropy.

[46] Blondel VD, Guillaume JL, Lambiotte R, Lefebvre E (2008). Fast unfolding of communities in large networks. J Stat Mech.

[47] Hagberg A, Swart PJ, Chult DA

[48] Goutte C, Gaussier E

[49] Qin T

[50] Sorower MS

[51] Wu J, Chen XY, Zhang H, Xiong LD, Lei H, Deng SH (2019). Hyperparameter optimization for machine learning models based on bayesian optimizationb. J Electron Sci Technol.

[52] Liashchynskyi P, Liashchynskyi P

[53] Feurer M, Hutter F

[54] Pedregosa F, Varoquaux G, Gramfort A, Michel V, Thirion B (2011). Scikit-learn: Machine learning in python. J Mach Learn Res.

[55] Chen T, Guestrin C

[56] Mason L, Baxter J, Bartlett P, Frean M (1999). Boosting algorithms as gradient descent. Adv Neural Inf Process Syst.

[57] Boehmke B, Greenwell B

[58] Medar R, Rajpurohit VS, Rashmi B

[59] Sarker IH (2021). Machine learning: Algorithms, real-world applications and research directions. SN Comput Sci.

[60] Nti I, Nyarko-Boateng O, Aning J (2021). Performance of machine learning algorithms with different K values in K-fold cross-validation. Int J Inf Technol and Comp Sci.

[61] Refaeilzadeh P, Tang L, Liu H

[62] Sechidis K, Tsoumakas G, Vlahavas I

[63] Szymański P, Kajdanowicz T (2017). A network perspective on stratification of multi-label data. Proc Mach Learn Res.

[64] Li W, Liu Y, Liu W, Tang ZR, Dong S, Li W, Zhang K, Xu C, Hu Z, Wang H (2022). Machine learning-based prediction of lymph node metastasis among osteosarcoma patients. Front Oncol.

[65] Tang Z, Wong HS, Yu Z (2024). Privacy-preserving federated learning with domain adaptation for multi-disease ocular disease recognition. IEEE J Biomed Health Inform.

[66] Chawla NV

[67] Chawla NV, Bowyer KW, Hall LO, Kegelmeyer WP (2002). SMOTE: Synthetic minority over-sampling technique. J Artif Intell Res.

